# The Importance of Research

**Published:** 1900-10-06

**Authors:** T. Clifford Allbutt

**Affiliations:** the opening of the Middlesex Hospital Medical School


### Hospital Clinics and Medical Progress.

### THE IMPORTANCE OF RESEARCH.

Address delivered by Professor T. Clifford Allbutt, F.R.S., at the opening of the Middlesex Hospital Medical School.

The October session of the Middlesex Hospital Medical School was opened on Monday, when the annual prize distribution took place. The ceremony was performed by Professor T. Clifford Allbutt, of Cambridge, who afterwards addressed the students. He said: I venture to think that our profession is only just waking up from centuries of routine and, even lately, from comparative slumber, and that there is a very great future, not merely before the profession itself but before any capable person who enters our ranks. Not many years ago—perhaps even within one's own remembrance—one turned on the pure fountains of youth, such as I see before me now, not into the fertile fields which they will enter now, but into a profession and into a state of science which was unfertile and which might almost be called a morass. You are entering now upon a profession which I venture to say has entirely altered not merely the routine of its daily life but which has entirely altered its outlook and the bases from which it works. When we are children we are imitative—I venture to say, for the moment, wholly imitative. As we grow older our thoughts generally turn back upon the past, and the young men who are worth anything enter upon an age of fascination for the past and an age of romance, which is generally followed by a stage in which those romantic feelings are carried into the future, in which they become Radical or Utopian or what they please. Then afterwards, having gone through those phases, they settle down to the serious business of life. That is a matter which causes all teachers the greatest anxiety—how will the man settle down in his future life? He may of course remain in any one of those stages. If so, he may be a little bit out of relation with practical life. But the tendency is very strong indeed to remain not in the Utopian stage, nor even to go back upon the romantic, but to drift back into the mere imitative stage of childhood; and if we look around we find that the tendency—and I speak from bitter experience, from the bottom of my own heart—is to drift back into the mere imitative stage of our youth and to go on using what is called our common sense, which I trust has its good side but which also has its imitative side of doing what others do all round us without considering how or why we do it. It appears to be of the utmost possible importance, then, that we should prevent you at this stage in your lives drifting back again to the imitative stage of infancy. I am sorry to say—or perhaps it is a good thing for the stability of society—that two-thirds of us will go back to sheer imitation. But there may be a precious remnant, some tentacles left to feel out into the future, inventively, and to try to feel something of the original life, to live lives of your own and not imitate the lives of those you see round about. How is it to be done? In regard to medical tradition I wish to speak with the greatest reverence, for without that we should have no medical capital to go upon, no social habits, no stability of any kind. That we all know. But how are we to teach you not to be imitative in your later lives? I am glad to hear that the best of all means has been taken of late in the Middlesex Hospital, namely, the establishment of means of research, not merely of research for each private member of the staff, which I trust no hospital is without, but such means of research that every single student may before he leaves these walls—after he has graduated, possibly—go through some short time of personal, and I will say original, research. I know that three months of research will probably not lead to your discovering anything of great importance in the world. But three months only of research—and six or twelve months would be much better—will lead to a discovery which is more valuable to you than many others: it will lead you to discover your own character and the inventive power within yourselves. It will do you no end of good, in fact, and, what is more, it will build up in you what we call a scientific habit of mind. A man never learns, unless he has been through the mill itself, how difficult it is to get the smallest particles of new knowledge. And yet the amount of positive knowledge which we possess is exceedingly small. What we treat patients upon is largely professional maxims and rules, which is not definite, positive knowledge at all—merely common sense and empirical maxims. But if you learn once for all in yourselves the enormous difficulty of getting at the bottom of anything in science, you get a scientific habit of mind, and learn what scientific manipulation and careful manipulation mean. It is a great pleasure to me to hear that these means of research are being multiplied in the Middlesex Hospital, and I trust they will be multiplied so that they may extend far beyond anything that, as far as I know, exists in any hospital to-day, in order that every one of you may have from three to six months of individual and personal research provided for you before you leave these walls.

One of the greatest misfortunes in the medical profession in the past, and one which we have by no means got rid of at the present day, is the tendency to remain content with abstract terms. It is easy enough to bring endless instances of such cases. For example, take the word "inflammation"—an abstract term, a word which has done perhaps more mischief than the whole of the weapons of war which have been manufactured in this country—the mere use of that word "inflammation." If you had asked anybody twenty-five years ago what he meant by "inflammation" he could only answer you in abstract terms; he knew nothing whatever about it, and yet he was using it as a basis for exceedingly active, and very often exeeedingly mischievous, treatment. And so things go on. Men read papers of all kinds—pages and pages—and do not in the least know what they mean. (Laughter.) It is all up in a balloon. Three months of research will knock that out of anybody. We must for convenience use abstract terms, but we must remember that in using them we have lost touch with facts and are talking up in the air. When you use such general expressions as "pneumonia" and the like, remember that although it is a convenience, it is a very dangerous convenience, and one which is taking you away from the case. The names of diseases need not be put aside, but we must remember how professional and hollow they are, and we must go back again to cases. When we deal with these cases we must remember that there is not such a thing as a general principle of therapeutic or of clinical medicine any more than there can be a general principle of biology—that our work must be tentative. A humble position think you? Perhaps, but still it is touching mother earth to restore our strength. Taking this particular case, looking at that particular man, we must realise that there never was a man like that before and never will be again. No two cases were ever alike any more than two leaves of a tree; so that we are discarding all these easy generalisations, and are putting them into the background, and what is coming up is cases and individuals. It is the rise of the patient that is the chief mark of modern medicine, and should be the aim of all medical systems.

The next question I should like to bring before you is concerning pathology and research, that peg of research being the peg upon which I should like it to revolve. Now, pathology has had an enormous development up to quite recently—and very great service it has done. But I think it has been felt for a great many years that there has been a tendency—one which, as far as I can see, must increase—for pathology to move away out of the wards. I think we cannot but feel that pathology will become more and more a disinterested science, and the professors of pathology will go out of practice, losing touch with the wards and becoming purely scientific investigators. But, you know, medicine is not a science; it is a practical art, and how we are to pluck our practical art out of the more and more distant department of pathology is, I think, another very great problem of the moment. If we do not do this, we shall fall again under the tyranny of abstract terms. How are we to do this? We are to do it, not by bringing the whole of the pathological department back, which is impossible, but by bringing certain portions of it back to the wards. I am not saying that the clinical laboratory ought actually to be within the four walls in which the patients are themselves, but what I do mean is that it must be on the same floor. It will not do to have a clinical laboratory in a wing. Secondly, the person in charge of the clinical laboratory—call him lecturer, demonstrator, or what you like—must have the free run of the wards himself. If the physicians and surgeons, if the clinical clerks, registrars, and so on, are to carry things out of the wards into the clinical laboratory, and then get the clinical laboratorian to work at them, they are detaching him from the clinical aspects of his subjects. He must himself have the free run of all the cases. He must not be told "This is from pneumonia," or "This is from enteric fever." They are abstract terms. He must know of the individual person it came from, and must know the case. I thus come back again to where I began—the rise of the patient and the rise of the case. This is only to be done by establishing in every hospital—not only where there are schools—a clinical laboratory at the door of the ward, where the functionary shall have the freest access to the patients themselves, being able to take what he pleases, to investigate what he pleases (within reasonable and humane consideration for the patients themselves, of course), and to do as any other physician would do, getting his own material and investigating it on the spot. One of the most melancholy things I know of is what happens to me frequently as I go into a country town. One goes to the hospital and finds everything in apple-pie order: most beautiful linen, the most lovely foods, charming nurses, and everything perfectly in order—excellent common sense, shrewdness and intelligence of a general kind in treating patients; but of medical science, absolutely nothing. The managers of the hospitals do not really care about the scientific part. They think medicine is taught you *en bloc*. You take it away from here, and if you are a shrewd, intelligent man you interpret it in your own way, and that is all you want. What has to be realised by them is that medicine is only just beginning, that we are on the very threshold. It is only by having active means of scientific investigation at the door of the bedroom in which the patient lies that you can expect to treat him according, not merely to the best lights of to-day, but according to the coming laws of to-morrow, and that is of the utmost possible importance. This really need not be a very ambitious affair. If we are brought to the facts I think we shall find that the apparatus at the most might cost £100. As regards the administrator, or whatever you may call the functionary, he would, of course, be a member of the resident staff. Such a man would want some physical means—means of testing blood pressure, and for making electric diagnosis, a chemical apparatus—not of a very extensive kind—a certain amount of bacteriological apparatus, and so on; but the whole thing could be got into a comparatively small compass. As regards the expense, in any county hospital where there was not a school a laboratory of that kind might soon be self-supporting. It would be a centre of illumination for all the clinical working men around. They would go there and be interested. They and their patients would gladly pay fees for the investigation.

